# Dietary Approach of Patients with Hormone-Related Cancer Based on the Glycemic Index and Glycemic Load Estimates

**DOI:** 10.3390/nu15173810

**Published:** 2023-08-31

**Authors:** Melpomeni Peppa, Aspasia Manta, Ioanna Mavroeidi, Constantinos Nastos, Emmanouil Pikoulis, Konstantinos Syrigos, Aristotelis Bamias

**Affiliations:** 1 Endocrine Unit, 2nd Propaedeutic Department of Internal Medicine, Research Institute and Diabetes Center, Attikon University Hospital, School of Medicine, National and Kapodistrian University of Athens, 12641 Athens, Greece; aspa.manta@gmail.com (A.M.); joannamavroeidi@gmail.com (I.M.); 2 3rd Department of Surgery, Attikon University Hospital, School of Medicine, National and Kapodistrian University of Athens, 12641 Athens, Greece; kosnastos@yahoo.gr (C.N.); mpikoul@med.uoa.gr (E.P.); 3 3rd Department of Internal Medicine, Sotiria Hospital, School of Medicine, National and Kapodistrian University of Athens, 11527 Athens, Greece; kostassyrigos.md@gmail.com; 4 2nd Propaedeutic Department of Internal Medicine, Research Institute and Diabetes Center, Attikon University Hospital, School of Medicine, National and Kapodistrian University of Athens, 12641 Athens, Greece; abamias@med.uoa.gr

**Keywords:** cancer, hormone-related cancer, carbohydrates, nutrition, diet, glycemic load, glycemic index, insulin resistance, nutrition, inflammation, oxidative stress, dietary advanced glycation end products

## Abstract

Hormone-related cancers, namely breast, endometrial, cervical, prostate, testicular, and thyroid, constitute a specific group of cancers dependent on hormone levels that play an essential role in cancer growth. In addition to the traditional risk factors, diet seems to be an important environmental factor that partially explains the steadily increased prevalence of this group of cancer. The composition of food, the dietary patterns, the endocrine-disrupting chemicals, and the way of food processing and preparation related to dietary advanced glycation end-product formation are all related to cancer. However, it remains unclear which specific dietary components mediate this relationship. Carbohydrates seem to be a risk factor for cancer in general and hormone-related cancers, in particular, with a difference between simple and complex carbohydrates. Glycemic index and glycemic load estimates reflect the effect of dietary carbohydrates on postprandial glucose concentrations. Several studies have investigated the relationship between the dietary glycemic index and glycemic load estimates with the natural course of cancer and, more specifically, hormone-related cancers. High glycemic index and glycemic load diets are associated with cancer development and worse prognosis, partially explained by the adverse effects on insulin metabolism, causing hyperinsulinemia and insulin resistance, and also by inflammation and oxidative stress induction. Herein, we review the existing data on the effect of diets focusing on the glycemic index and glycemic load estimates on hormone-related cancers.

## 1. Introduction

Hormone-related cancers (HRCs) constitute a group of cancers dependent on hormone levels that play an essential role in cancer growth. Breast (BC), endometrial (EC), ovarian (OC), cervical (CC), prostate (PC), and testicular cancers (TC) are referred to as HRCs based on the similar hormone-dependent pathogenetic mechanisms of tumorigenesis [1]. Recent studies support that thyroid cancer (ThC) is also another HRC, as it shares similar pathogenetic pathways [2].

**Figure 1:**
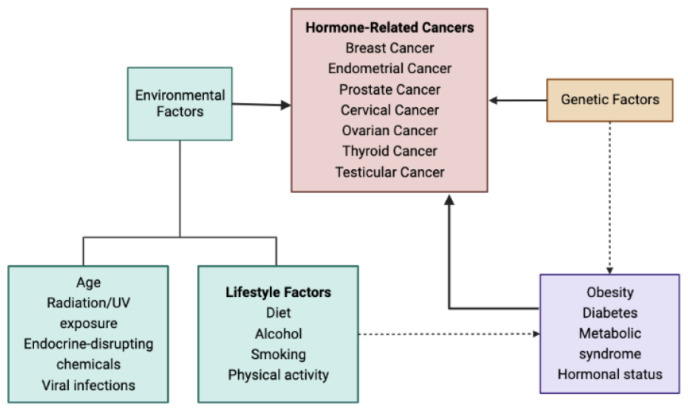
Risk factors for hormone-related cancers.

Even though HRCs are the leading cause of death in many countries [3], their pathogenesis has yet to be fully clarified. Age, race, longtime exposure to estrogens or androgens, radiation, and a positive family history are well-known risk factors [4]. Obesity alone or as part of the metabolic syndrome also seems to be related to HRC development and prognosis [5]. The different risk factors associated with HRCs are presented in Figure 1.

Beyond these widely recognized risk factors, diet is an important environmental factor and an epigenetic modifier involved in the steadily increased prevalence of various diseases, including cancer [6]. Through the production of oxidative stress (OS), inflammation, and insulin resistance (IR), dietary involvement impacts cancer growth, either directly or indirectly [7].

Dietary plans based on a Westernized lifestyle trigger obesity and metabolic syndrome but can also directly affect hormonal homeostasis, leading to cancer [8]. Beyond general diet characteristics, individual dietary patterns and certain food constituents can influence gene expression, protein synthesis, cell signaling, and other essential carcinogenesis events [9], but it remains unclear which specific dietary components mediate this relationship. 

The exposure of food production and processing to endocrine-disrupting chemicals is an important risk factor for cancer, particularly HRCs [10,11]. Additionally, dietary advanced glycation end products (dAGE), formed by food processing and preparation methods, are involved in certain aspects of health and disease, including cancer [12,13,14]. Dietary AGE has been related to OS, inflammation, aging, and cellular dysfunction, all of them leading to cancer development and progression [15,16,17,18].

Carbohydrate (CHO) intake is also considered a modifiable risk factor for cancer [19]. CHO consumption raises blood insulin and glycemia to variable degrees, depending on CHO type amount, processing method, and availability of other nutrients [20].

At present, one of the primary cancer therapy modalities is considered to be lifestyle changes. The World Cancer Research Fund and the American Institute for Cancer Research (WCRF/AICR) recommend a healthful diet as one of the most important modifiable risk factors for decreasing cancer risk [21]. The recommendations support the intake of mostly plant-based foods, and special emphasis has been given to the way the food is processed and cooked. Some balanced dietary patterns, such as the Mediterranean diet, could contribute to the dietary prevention of Western diseases [8]. Such diets constitute a crucial therapeutic intervention in cancer patients, reducing the risk of aggressive disease and disease progression [22]. Dietary modifications in the form of developing low-dAGE diets have also been found beneficial, following the publishing of a list identifying the AGE content of various common foods [16]. Additionally, modifying CHO intake may be one of the potential ways to prevent cancer, enhance quality of life, and even alter the course of the disease [19].

A low-CHO diet is used in order to starve cancer cells while also normalizing plasma insulin levels. Indeed, a typical low-CHO diet, the ketogenic diet, has been applied in cancer treatment [23].

To further explore the relationship between CHO and cancer, many studies use two important estimates, the glycemic index (GI) and glycemic load (GL), which express the impact of dietary CHO on body homeostasis [24].

The GI reflects the effect of any CHO-rich food on postprandial blood glucose response, compared to an equivalent CHO portion of a reference food (white bread or glucose). Higher rates of CHO absorption result in higher postprandial glucose and GI values, which are also affected by the type of CHO and the way and extent of food processing [25]. The GL reflects the effect of total CHO consumption on blood glucose responses, expressed as the percentage of a food’s GI and total available CHO content divided by 100. GL considers both intakes, the CHO content, and the total glycemic effect, indicating the total insulin demand. It is, thus, considered a better measure than GI for characterizing the glycemic effect of a diet [26]. Although GI and GL are indexes of the effect of CHO on the total body metabolism, the overall GI reflects the quality of CHO consumption, whereas the total GL reflects both the quantity and quality of CHO consumption [27].

**Figure 2:**
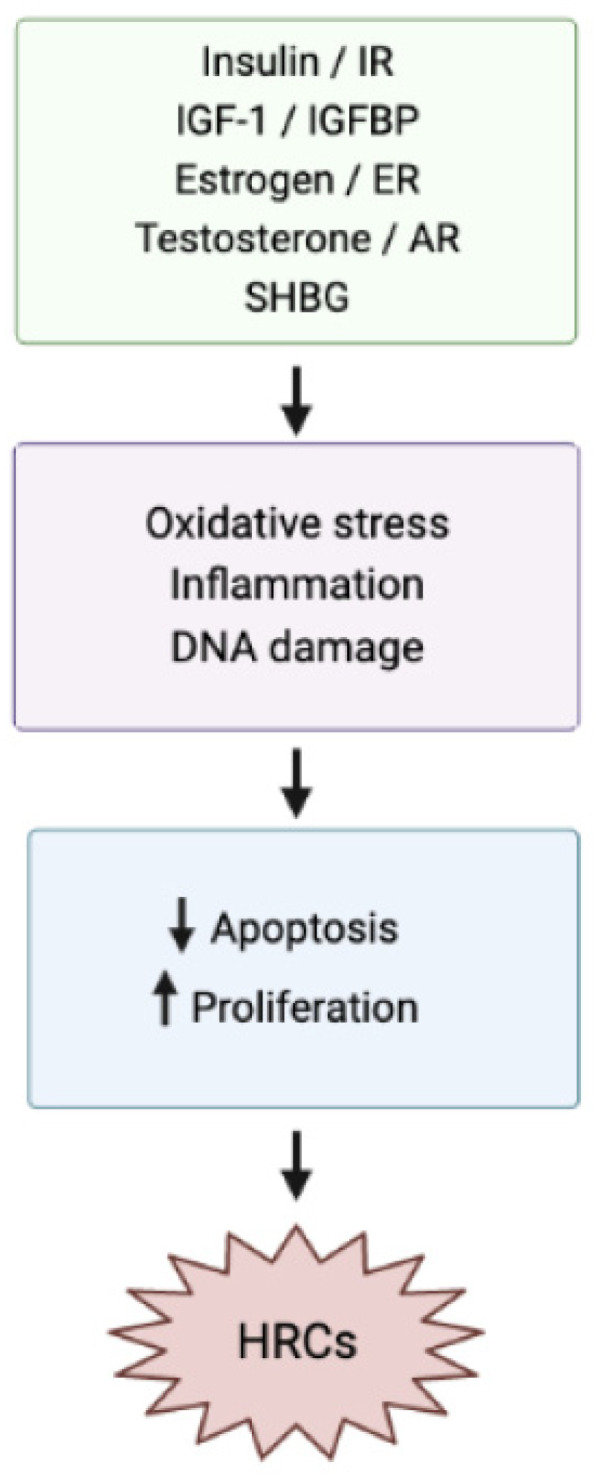
Basic pathophysiological pathways of hormone-related cancers. IR: Insulin Resistance; IGF-1: Insulin-like Growth Factor-1; IGFBP: Insulin-like Growth Factor Binding Proteins; ER: Estrogen Receptors; AR: Androgen Receptors; SHBG: Sex Hormone-Binding Globulins; HRCs: Hormone-Related Cancers (The arrow~↓~means decrease, while the arrow~↑~means increase.).

High GI and GL diets have been associated with hyperinsulinemia and insulin resistance (IR), leading to metabolic syndrome, obesity, diabetes, cardiovascular disease, and cancer [28]. Obesity-associated proinflammatory adipokines, such as leptin, interleukin-6, and tumor necrosis factor α, suppress normal insulin signaling, contributing to IR [29]. Insulin mediates cancer development not only through direct effects on cells but also through indirect effects on insulin-like growth factors (IGFs) and IGF-binding proteins (IGFBPs), increasing the bioactivity of IGF-1 [25]. The net result is inhibition of apoptosis, stimulation of cell proliferation, angiogenesis, sex-steroid hormone synthesis, and thus increased levels of free estrogen and testosterone levels, which bind to mutated estrogen/androgen receptors, increasing the risk of HRCs [30]. Adipokine-mediated chronic inflammation causes cellular stress, which is associated with enhanced genetic instability and DNA damage [29]. Tumor-associated adipocytes that are part of the tumor microenvironment may also play a role in carcinogenesis, as indicated by research on non-HRCs [31]. The basic underlying pathophysiological mechanisms of HRCs are presented in Figure 2.

In the context of dietary modifications as one of the therapeutic approaches in patients with cancer, dietary plans that take into account the GI and GL indexes can be created using available lists that give information on the serving sizes, CHO content, GI and GL indexes of a variety of meals [32].

The aim of the present review is to present the existing literature and compile all relevant information on the effect of diets, taking into account the GI and GL estimates on HRCs.

## 2. Effects of Dietary GI and GL Indexes on HRCs

### 2.1. Effects of Dietary GI and GL Indexes on Breast Cancer

Breast cancer (BC) is the second most common cancer in women after skin cancers and the second highest cause of death after lung cancer. BC is the leading cancer type in obese women, and increased awareness of this relationship has led to much effort to prevent obesity as a cause of BC [33]. Healthy dietary patterns and weight loss interventions focusing on abdominal adiposity are related to a lower risk and better prognosis of BC, a lower risk of BC recurrence, and reduced all-cause mortality. Nevertheless, there is currently little information about BC’s underlying mechanisms, the proper dietary manipulation, and the most effective dietary pattern for weight management and cancer development and prognosis [33,34].

Dietary caloric intake and total CHO intake have been linked to BC. Simple sugar, sucrose, maltose, and fructose were positively associated with BC [35]. High total sugar intake, especially added sugar, sucrose, and fructose, as well as CHO from fruit juice after a BC diagnosis, were also associated with poorer prognosis [36].

High GL and GI diets have been extensively studied in relation to the development of BC, with conflicting results due to research on different and heterogeneous populations. However, most research supports that high GI and GL dietary patterns are related to BC development and prognosis. Some studies have identified only GI or GL as better correlates with the BC risk in different subgroups of women.

Premenopausal women: Several studies have shown a significant association of GI and GL indexes with BC risk in overweight [37,38], lean (BMI < 25) [39], or irrespective of BMI [40] premenopausal women, as well as in those with low levels of physical activity [41]. The association of only GI with increased BC risk was found by Sasanfar et al. [42]. When hormone receptor status was examined, Woo et al. reported this association for the ER+ or PR+ type [43]. Amadou et al. found no association of BC risk with GI and GL but a strong correlation with the total CHO intake in overweight premenopausal women [44]. Dietary intervention with increased consumption of low GL diet significantly affected several miRNAs related to various cancer pathways in healthy premenopausal women with a high BC risk [45]. A retrospective study on dietary habits during adolescence discovered that a higher dietary GI was associated with an increased risk of BC later in life [46]. In regard to CHO, higher-quality CHO intake was related to a lower risk of BC in premenopausal women [42,47].

Postmenopausal women: High GI and GL have been associated with an increased risk of BC in postmenopausal women, as well [43,48,49,50,51]. Lajous et al. noted that particularly overweight women and women in the greatest waist circumference subgroup were more prone to BC when following a high GI and GL diet [48], while Silvera et al. noted this association mainly in normal-weight women [52].

Concerning the BC receptor subtype, different studies in postmenopausal women had varying results. The link between GL and GI and BC risk was noted in the ER+/PR- BC subtype [49], in the ER-BC subtype [48,53], in the ER-/PR-BC subtype [54], as well as in all subgroups of hormone receptor status [43]. Evidence also links GL to in situ BC [55]. Among postmenopausal women with vegetable intake below the median (307 g/d), elevated dietary GI was also linked to an increased risk of BC [50].

High GL was also directly associated with high mammographic breast density [56], a well-recognized risk factor for BC that is inversely associated with BC-specific survival [57]. Dietary intervention with a low GL diet in healthy postmenopausal women with a mammographic density >50% resulted in a significant decrease in BC risk [58].

Any menopausal status: High GI and GL were linked to an increased BC risk independently of menopausal status, physical activity, or weight [59,60]. In a recent study that examined a large cohort of stage I–III BC patients, higher post-diagnostic GI and GL were associated with a higher risk of all-cause mortality and BC-specific mortality, respectively [61]. GL alone [62] or GI alone [63,64] were also correlated to an increased risk of BC regardless of menopause status. A large prospective study that examined adiposity-related cancers found that a low-GI diet was associated with a 49% lower BC risk [65]. The investigation of the effect of diet on different molecular BC subtypes revealed that only GI was positively correlated with luminal A (RE+ and/or PR+/HER2-), HER2+ (RE+ and/or PR+/HER2+ and RE-/PR-/HER2+) and triple negative (TN) (RE-/PR-/HER2-) BC [66].

Several studies have focused on the “Diabetes Risk Reduction Diet” (DRRD), which is, by definition, a diet characterized by low GI foods. Although this diet was inversely associated with the risk of type 2 diabetes [67], a number of studies supported that a greater DRRD adherence was associated with reduced BC risk [68,69], especially in normal-weight and postmenopausal women [70], but also with improved survival outcomes in BC patients [71].

Despite the positive correlation noted by a great number of studies, some studies report no significant association between GL or GI and the risk for BC in premenopausal women [72], postmenopausal women [73,74,75], or regardless of menopausal status [76,77].

### 2.2. Effects of Dietary GI and GL Indexes on Endometrial Cancer

Endometrial cancer (EC) ranks as the 15th most frequent cancer overall and the sixth most frequent cancer in women [78]. Hormonal imbalances in premenopause and menopause, namely increased estrogens/low progesterone levels, polycystic ovarian syndrome, obesity, hyperinsulinemia, IR, physical inactivity, type 2 diabetes, and hypertension, are all connected to EC [79]. More than half of EC cases are currently attributable to obesity, which is recognized as an independent risk factor. This association follows a dose–response relationship, with the incidence of EC increasing as body mass index (BMI) increases [29].

The Mediterranean diet has been proven beneficial in several aspects of gynecological health [80], whereas a diet high in complex CHO causes hormonal imbalance that leads to obesity and many other diseases, including cancer [81]. Several in vivo and in vitro studies also support that a high GL diet over an extended period causes hyperinsulinemia and IR [82].

The quantity and quality of CHO may contribute to the etiology of EC. Consumption of CHO, specifically total sucrose intake and complex CHO intake, was associated with an increased risk of EC [83].

The positive association between GI and GL with the risk of EC has been shown in studies mainly undertaken in Western countries with a high incidence of high GI and GL diets. This correlation was found to be dependent on menopausal status, body size, or physical activity [84,85,86,87,88,89]. However, a study in Japan, where individuals have different dietary habits and lower BMI, found null associations among GI, GL, and the risk of EC [78]. In addition, several case–control studies found no connection between dietary CHO intake, GI and GL, and EC risk [78,82,90,91,92]. 

The impact of high GI and GL diets seems to be evident in obese pre- or postmenopausal women, especially under hormone replacement treatment [93]. Xu et al. discovered that consumption of high GL or GI meals, but not just regular CHO, may also raise the risk for EC in lean and normal-weight women [87]. Other studies observed a stronger correlation between GI but not GL and EC in obese or older women with greater BMI and those on hormone replacement treatment [84]. The Australian National Endometrial Cancer Study also showed that GI but not GL was linked to an increased risk for EC [82].

Galeone et al.’s meta-analysis supported an elevated risk for EC with high GL but not GI [94]. Similar findings were found in studies in non-diabetic and obese women with low levels of physical activity [85,86]. In contrast with the above data, Coleman et al. found that high CHO and GL diets are preventative measures against the onset of EC [92].

### 2.3. Effects of Dietary GI and GL Indexes on Prostate Cancer

Prostate cancer (PC) is the second most frequent malignancy in men [95]. Age, family history, race, and ethnicity are recognized risk factors for PC. Other less-known factors include IR, obesity, metabolic syndrome, and alcohol use [96]. Multiple studies have shown that there is a link between obesity, excessive body fat, and PC, especially the aggressive variant [97].

Various studies have examined the hypothesis that reducing CHO may slow PC growth by lowering serum insulin or altering the insulin-like growth factor (IGF) that has shown mitogenic and antiapoptotic effects on prostate epithelial cells. Animal studies showed that a no-CHO ketogenic or a low-CHO diet may slow prostate tumor growth. In humans, only one study found a high intake of refined CHO associated with increased PC risk, but others did not confirm this hypothesis [98], finding no significant associations between a high intake of refined CHO and the risk of PC [99].

Macronutrients, such as refined CHO, especially high intake of cake, biscuits, pasta, and rice, as well as processed meat, milk, dairy products, and some micronutrients like calcium, lycopene, selenium, and vitamin E, are all related to PC risk [30]. This is further supported by the observation that immigrants from Asia and Africa to Western nations have higher incidence rates of PC, partially mediated by the changes in dietary habits and a different lifestyle inducing IR [100]. A higher dietary fat intake is also correlated with higher PC mortality rates [101].

There is conflicting epidemiological evidence about the contribution of dietary GI, GL, and total CHO intake to PC risk. Although it can be challenging to pinpoint the causes of inconsistencies, significant variations in dietary practices may play a role, as well as race and genetic factors [65,102].

Experimental studies have shown that a low-fat/low-GL diet and the resulting weight loss are linked to several alterations in gene expression, affecting growth, metabolism, and redox in prostate epithelial cells [103]. Moreover, high-fat/low-GI and extremely low-fat vegan diets showed no impact on tumor biology, as measured by changes in tumor gene expression [96,104].

Some clinical studies have demonstrated a direct correlation between dietary GI and GL and the risk of PC [25,30,102,104]. A positive dose–response relationship between only GI and PC has been identified [105,106,107], with GI playing a role in more aggressive diseases [107]. However, other studies have failed to uncover any meaningful link [30,65,100,108].

More research needs to be performed to clarify the effects of dietary manipulation regarding GI GL and PC risk and give further insight into the underlying mechanisms.

### 2.4. Effects of Dietary GI and GL Indexes on Ovarian Cancer

Ovarian cancer (OC) is the fifth most frequent cancer worldwide and one of the most lethal female gynecological malignancies [109]. Chronic hyperinsulinemia can develop from long-term CHO consumption, and it has been claimed that hyperinsulinemia may raise the risk of OC by activating numerous pathways that include insulin-like growth factor 1 (IGF-1) [110,111].

Although the role of obesity in OC is still unclear [112,113], recent research suggests that body composition, namely high adiposity and sarcopenia, may impact OC outcomes [114]. Chronic inflammation has been suggested as an underlying mechanism contributing to ovarian carcinogenesis by stimulating DNA damage and promoting enhanced cell division, which can lead to DNA repair abnormalities, promoting angiogenesis, and facilitating invasion. Long-term use of proinflammatory foods such as saturated fat, CHO, and animal proteins increases the risk of OC [115]. Consumption of greater quality macronutrients, such as carbohydrates, fats, and proteins, has also been linked to improved survival in OC patients [116]. Total consumption of CHO, complex CHO, but mostly sugar, has been linked to OC in obese patients [117].

Few studies have investigated the effect of a high GL/GI diet on OC. Most of them have shown a link between GL and OC, either in overweight/obese women [117] or irrespective of BMI [118]. Silvera et al. found that GL was related to a 72% increase in the risk of OC, with the connection being somewhat stronger in postmenopausal women [119].

A clear relation was also observed between GI, GL, and OC, regardless of menopausal state [120]. GI prior to OC diagnosis was strongly associated with shorter survival in a large cohort of women with invasive OC [121]. A large cohort study in postmenopausal women, on the other hand, found an inverse relationship between GI/GL and OC after a 12-year follow-up. These findings contradict our expectations and warrant additional investigation [122].

### 2.5. Effects of Dietary GI and GL Indexes on Cervical Cancer

Cervical cancer (CC) is currently the fourth most frequent cancer type in women worldwide and is associated with several environmental and lifestyle risk factors [123]. Among them, obesity affects both screening results and overall survival in CC patients [124].

Even though both a high-CHO diet [125,126] and plasma glucose levels [127] have been associated with an increased risk of CC, dietary GL was linked to an increased incidence of CIN1 but not of CIN2/3 or CC. This correlation was strongest among women with a BMI < 23, premenopausal, or HPV-positive [128].

### 2.6. Effects of Dietary GI and GL Indexes on Thyroid Cancer

Thyroid cancer (ThC) is the most common endocrine malignancy, with a clear upward trend in incidence over the last decades and several acknowledged risk factors [129]. Obesity and high waist circumference have both been associated with an increased risk for ThC, possibly via chronic inflammation and the production of various cytokines and adipokines [130]. A series of case–control and prospective studies have consistently found a link between obesity and thyroid cancer risk. Research on the effect of dietary habits in ThC has revealed a protective effect of a diet rich in fruits and vegetables in different populations [131,132,133], while diet-associated inflammation is potentially associated with an increased risk for ThC [134]. The mechanisms underlying the link between CHO and differentiated ThC (DTC) are not fully understood, but we could hypothesize that, as with other HRCs, the same actions of insulin and IR could be associated with an increased risk of DTC in individuals consuming high-CHO diets [135].

Regarding GL and GI, there is a scarcity of studies examining this relationship. A large prospective cohort study with a mean follow-up of 11 years indicated that excessive starch and GI diet intake increased the risk for differentiated ThC in patients with BMI ≥ 25 [136]. Another case–control study found that high levels of GI and GL are linked to greater ThC risk, with follicular ThC exhibiting a slightly higher risk for high levels of GL compared to papillary ThC [137].

### 2.7. Effects of Dietary GI and GL Indexes on Testicular Cancer

Testicular cancer (TC) is a relatively uncommon cancer affecting young men [138]. Current evidence does not support that the pathogenesis of testicular cancer is related to obesity [139].

Excessive fat consumption and dairy products have been associated with an elevated risk of TC [140]. However, according to most recent research, there is no reliable indication to support this assumption, and diet does not seem to influence TC risk [141,142]. Overall, the association remains unclear and requires further research.

## 3. Conclusions

The increased prevalence of cancer, particularly HRCs, poses various dilemmas in terms of prevention and therapeutic interventions. In addition to the classical risk factors, other less traditional ones, such as dietary composition and patterns, as well as exposure to sterilization and processing or cooking methods, seem to be involved in cancer development and progression. CHO intake is considered a modifiable risk factor for cancer in terms of the beneficial effect of consuming low CHO diets. GI and GL are well-known estimates of the effects of CHO on total body homeostasis. High GI and GL diets appear to induce oxidative stress, IR, growth factors, obesity, and diabetes mellitus, all related to cancer, including HRCs. On the contrary, low GI and GL diets seem to prevent cancer in general especially HRCs. However, there are some limitations as the provided evidence is based on epidemiological studies that can be influenced by multiple factors and fails to provide a comprehensive understanding of the interactions between diet, CHO, GI-GL, metabolism, and tumor development. It is obvious that more research needs to be performed with prospective studies in order to clarify these associations. The existing data further support that GI and GL should be included in the dietary approach of patients with cancer in general and HRCs in particular, either for prevention or treatment.

## 4. Future Directions

Future research in the context of diet and HRCs holds immense promise in advancing our understanding of the complex interplay between diet, hormones, and cancer development. As we delve deeper into this field, researchers are increasingly focusing on unraveling the intricate mechanisms by which dietary factors and hormonal imbalances contribute to cancer risk. 

With advancing technology and improved study designs, researchers are exploring further the complex interplay between GI, GL, and HRCs. Future studies will likely focus on elucidating the underlying mechanisms through which high-GI and high-GL diets influence hormonal pathways, IR, and inflammation, all of which are known to play crucial roles in cancer development and progression. Furthermore, large-scale epidemiological and prospective cohort studies will enable the evaluation of long-term dietary patterns and their impact on HRCs. The integration of genomic and metabolomic profiling will provide valuable insights into individual susceptibility and the molecular pathways involved.

By understanding the relationship between GI, GL, and HRCs more comprehensively, future research may pave the way for tailored dietary interventions, risk stratification, and targeted therapeutic strategies, ultimately leading to improved patient outcomes and a reduction in the incidence of hormone-related cancers.

## Data Availability

Data supporting this article are included within the reference list.
